# Photothermal Conversion Profiling of Large-Scaled Synthesized Gold Nanorods Using Binary Surfactant with Hydroquinone as a Reducing Agent

**DOI:** 10.3390/nano12101723

**Published:** 2022-05-18

**Authors:** Thabang Calvin Lebepe, Oluwatobi Samuel Oluwafemi

**Affiliations:** 1 Department of Chemical Science, University of Johannesburg, Johannesburg 2028, South Africa; calvyn.tl@gmail.com; 2 Centre for Nanomaterials Sciences Research, University of Johannesburg, Johannesburg 2028, South Africa

**Keywords:** gold nanorods, hydroquinone, binary surfactant, aspect ratio, photothermal efficiency

## Abstract

Photothermal application of gold nanorods (AuNRs) is widely increasing because of their good photothermal conversion efficiency (PCE) due to local surface plasmon resonance. However, the high concentration of hexadecyltrimethylammonium bromide used in the synthesis is a concern. Moreover, the mild and commonly used reducing agent-ascorbic acid does not reduce the Au(I) to A(0) entirely, resulting in a low yield of gold nanorods. Herein we report for the first time the PCE of large-scaled synthesized AuNRs using the binary surfactant seed-mediated method with hydroquinone (HQ) as the reducing agent. The temporal evolution of the optical properties and morphology was investigated by varying the Ag concentration, HQ concentration, HCl volumes, and seed solution volume. The results showed that the seed volume, HQ concentration, and HCl volume played a significant role in forming mini-AuNRs absorbing in the 800 nm region with a shape yield of 87.7%. The as-synthesized AuNRs were successfully up-scaled to a larger volume based on the optimum synthetic conditions followed by photothermal profiling. The photothermal profiling analysis showed a temperature increase of more than 54.2 °C at 2.55 W cm^−2^ at a low optical density (OD) of 0.160 after 630 s irradiation, with a PCE of approximately 21%, presenting it as an ideal photothermal agent.

## 1. Introduction

The applications of Gold nanorods (AuNRs) have grown widely in different fields, from biomedical to sensing and electronics applications [1,2]. AuNRs can absorb photons at different spectral regions by merely adjusting their aspect ratio, which puts them at a better advantage for different applications than other gold nanostructures [3,4,5]. Various methods of synthesizing AuNRs have been established, such as the template method [6,7,8], electrochemical method, photochemical method [9,10], and seed-mediated method [5,11,12,13,14]. However, some challenges associated with these methods include low product yield, robustness to minor impurities, precise control over AuNRs’ surface chemistry, stability, and most importantly, a feasible method to suit specific applications. Factors such as temperature [15,16], pH [14,17,18,19,20], type of surfactant [5,13,14,19,21,22,23,24,25,26], reagent concentration [10,27,28,29,30,31,32,33], additives [34,35,36], and the seed quality [11,12,13,14,37,38] have been shown to impact the growth and purity of AuNRs [1,2]. Currently, the seed-mediated method is the most used method to synthesize AuNRs. In a typical seed-mediated method, 0.1 M hexadecyltrimethylammonium bromide (CTAB) is usually used; however, this concentration contributes to the AuNRs’ high cytotoxicity, which hinders its biological application [9,14,19,39,40].

Nikoobakht and El-Sayed reduced the CTAB concentration by combining it with benzyldimethylammoniumchloride (BDAC) and obtained AuNRs with larger aspect ratios (>5) [37]. Other researchers have shown that monodispersed AuNRs with tunable dimensions can be obtained by combining CTAB with sodium oleate (NaOL) [13,14,40]. The NaOL functions as both a capping agent and a reductant [13,14,19,40]. Furthermore, similar to the single-surfactant method, the AuNRs’ length, diameter, and LSPR peak can be tuned by varying the pH of the growth solution, Ag concentration, and seed solution [13,14,40]. The most common reducing agent in binary surfactants is ascorbic acid. However, the weaker reducing agent hydroquinone (HQ) has never been used for binary surfactants in the seed-mediated method. HQ has been used in single-surfactant seed-mediated methods, and it has been shown to produce AuNRs with high shape monodispersity yields of above 95% and reduction yields of nearly 100%, which is quantitatively better than Ascorbic acid [20]. Thus, we proposed the use of HQ as a reducing agent. Herein, we report, for the first time, the synthesis of AuNRs using the binary surfactant (CTAB and NaOL) seed-mediated method and HQ as the reducing agent. The effect of Ag concentrations, HQ concentrations, HCl volume, HCl concentrations, and the seed solution volume on the shape and aspect ratio of the as-synthesized AuNRs was investigated using UV-Vis-NIR spectroscopy and TEM. The as-synthesized AuNRs photothermal conversion efficiency was also evaluated. We further demonstrated that AuNRs synthesized from the binary surfactant seed-mediated method using HQ as the reducing agent can be scaled up to produce a similar yield as the small-scale synthesis. In addition, the as-synthesized AuNRs had a photothermal conversion efficiency of approximately 21%, which makes them an ideal photothermal agent for photothermal application in cancer and other bio-applications.

## 2. Materials and Methods

### 2.1. Materials

Hydrogen tetra-chloroauric hydrate (HAuCl_4._ xH_2_O, 99.9%), sodium borohydride (NaBH_4_, 99%), silver nitrate (AgNO_3_, 99%), cetyltrimethylammonium bromide (CTAB, ≥99%), Hydroquinone (HQ, 99%), sodium oleate, (NaOL, ≥99%), and hydrochloric acid (HCl, (12.1 M)) were purchased from Sigma-Aldrich, Kempton Park, South Africa. All glassware used in the experiments was cleaned, washed thoroughly with MilliQ water (15.0 MΩ cm @ 25 °C), and dried before use.

### 2.2. Synthesis of Gold Nanorods

The synthesis was initialized by preparing fresh stock solutions of 0.10 M and 4.00 mM AgNO_3_ and hydroquinone (0.10, 0.082, and 0.064 M). The seed solution was prepared as follows: 0.346 g of CTAB was added to 9.5 mL of warm deionized water (DW) and stirred to dissolve the powder completely. After cooling the CTAB solution to room temperature, 0.5 mL of the HAuCl_4_·xH_2_O (0.01 M) solution was added and mixed gently for 15 min, followed by the addition of 0.46 mL freshly prepared ice-cold NaBH_4_ (0.1 M) in NaOH (0.1 M). The solution was vigorously stirred for 2 min to produce a light brown solution, which serves as the seed solution. This solution was left undisturbed for over 30 min before use. The binary surfactant solution of CTAB (0.037 M, 7 g) and NaOL (0.23 M, 1.234 g) was prepared in 250 mL of warm DW. Furthermore, 0.50 mL of 0.01 M HAuCl_4_·xH_2_O was added to 8 mL of the previously prepared binary solution and stirred for 15 min. Then, 40 μL of AgNO_3_ (0.1 M) and 12.1 M HCl solutions were introduced to the solution under gentle stirring. Finally, 0.50 mL of different concentrations of hydroquinone (HQ) were added under continuous stirring, followed by the addition of different volumes of the seed solution. The solutions were left uninterrupted for 16−20 h at room temperature, followed by purification and characterization.

### 2.3. Synthesis of Gold Nanorods in Large Scale

The large-scale synthesis of the AuNRs solution (500 mL) was prepared by preparing a seed solution of 100 mL with similar concentrations as above and left undisturbed for 30 min before use. The growth solution was prepared by mixing the binary surfactant solution (CTAB 0.037 M and NaOL 0.23 M in 400 mL of warm DW) with 100 mL of 0.01 M HAuCl_4_·xH_2_O, followed by 2 mL of AgNO_3_ (0.1 M) and 1.8 mL HCl (12.1 M) under gentle stirring. Finally, 25 mL HQ (0.1 M) and a 100 mL seed solution were added under continuous stirring. The solution was left uninterrupted for 16−20 h at room temperature, followed by purification and characterization.

### 2.4. Photothermal Profiling of AuNRs

Power density optimization was performed by placing 3 mL of deionized water in a UV-Vis quartz cuvette followed by irradiation under a continuous 808 laser at different power densities (1.27, 2.55, 3.82, and 5.09 W·cm^−2^). The temperature changes were measured using an RS-1384 PRO thermocouple. The photothermal efficiency of AuNRs was measured by placing 3 mL of AuNRs solutions into a UV-Vis quartz cuvette followed by irradiation with two different power densities (1.27 and 2.55 W·cm^−2^) separately. The temperature changes were measured using the RS-1384 PRO thermocouple and the FLIR E4 thermal camera. The photothermal conversion efficiency (PCE) was evaluated by irradiating 3 mL AuNRs with 808 nm power density of 1.27 W·cm^−2^ for 10 min, and then the laser system was switched off. The temperature of the dispersions was recorded every 30 s using a thermocouple. The PCE was calculated following Li et al.’s calculation with modifications [41].

### 2.5. Characterization Techniques

The as-synthesized AuNRs UV-Vis-NIR absorption spectra, morphology, and hydrodynamic dimension were obtained using a UV-Vis-NIR JASCO V-770 spectrophotometer (JASCO Corp., Tokyo, Japan), high-resolution transmission electron microscopy (HRTEM, JEOL 2010, 200 KV, Tokyo, Japan), and Microtrac MRB’s NANOTRAC Wave II (Microtrac MRB, Duesseldorf, Germany), respectively. The particle size distributions and aspect ratios of the AuNRs were measured from TEM images using ImageJ software. A dst11-LUMICS-808 nm 27W continuous Nd: YV04 air-cooled laser system (OsTech e. K., Berlin, Germany) with an optical fiber to deliver an 8 mm beam diameter was used for the irradiation.

## 3. Results

**Figure 1:**
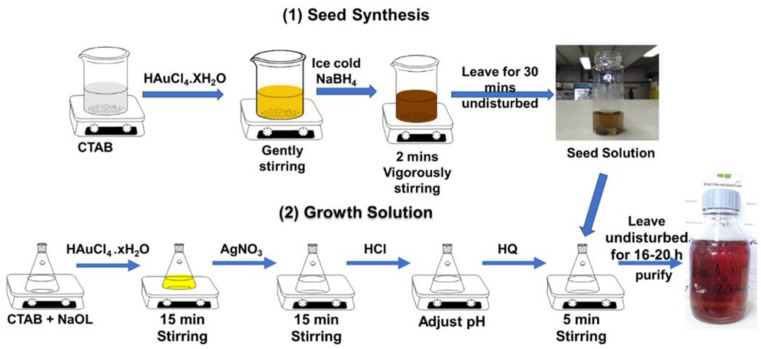
Schematic representation of the AuNRs’ synthesis procedure of seed-mediated binary surfactant with hydroquinone.

The synthesis of AuNRs with a binary surfactant using hydroquinone as a reducing agent was successfully executed via the binary surfactant seed-mediated method, as illustrated in Figure 1. The synthesis was established using a typical seed-mediated method by separating the nucleation reaction from the primary growth solution [37]. The seed solution was prepared by reducing Au(III) to Au(0) with ice-cold NaBH_4_ in NaOH. The NaOH slowed the reaction rate during the nucleation of the Au in the seed solution. The binary surfactant mixture of CTAB and NaOL was used as the growth solution. We investigated the effect of HQ concentrations, HCl volume, and seed solution volume on the AuNRs’ growth. These factors have been reported previously in different AuNRs’ syntheses to play a significant role in the AuNRs’ formation, especially the seed-mediated method [14,20,40]. The NaOL in the binary surfactant mixture has been reported to be responsible for reducing the Au^3+^ to Au^1+^ in the early stage of the experiment, unlike in the single surfactant due to the double bonds on the long-chain structure of NaOL [14]. The reduction was observed in the reaction after the addition of AgNO_3_, indicated by a color change from yellow to colorless before the HQ addition.

**Figure 2:**
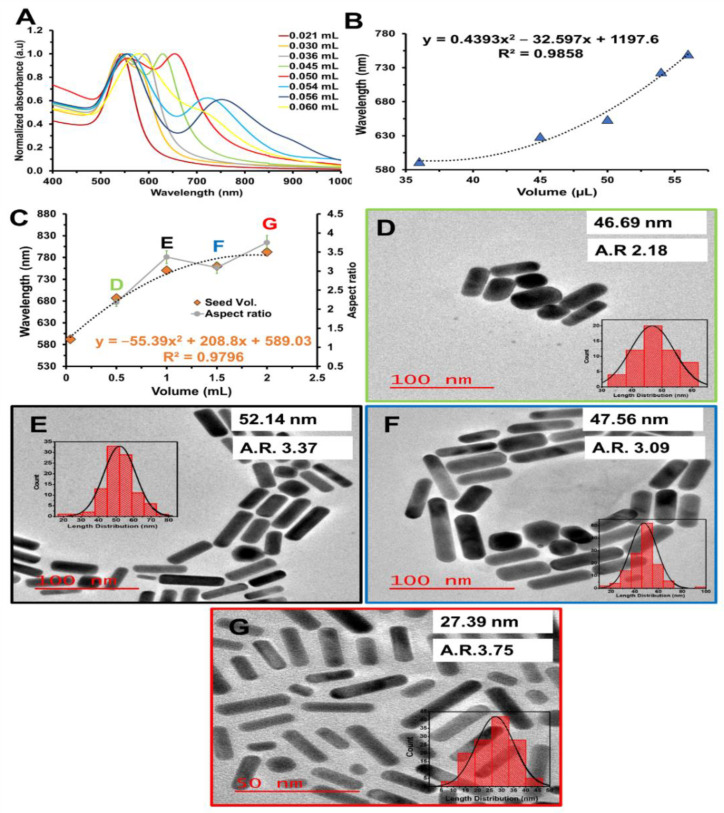
(**A**) Normalized UV-Vis-NIR spectra of AuNRs at different HCl volumes (**B**) Second-order polynomial relationship between the HCl volume and the corresponding LSPR band wavelengths. (**C**) The relationship between the seed volume against the corresponding LSPR band wavelengths and aspect ratio. TEM image of AuNRs synthesis with (**D**) 0.5, (**E**) 1.0, (**F**) 1.5, and (**G**) 2 mL of seed solution, while other parameters were kept constant.

The as-synthesized AuNRs were characterized using UV-Vis-NIR spectroscopy and TEM. It is known that HQ is acid-dependent [20], therefore we first investigated the effect of pH by adding different HCl volumes (0.021, 0.030, 0.036, 0.045, 0.050, 0.054, 0.056, 0.060 mL), while keeping the AgNO_3_ concentration, HQ concentration, and seed solution volume constant. Figure 2A shows the UV−vis−NIR spectra of the as-synthesized AuNRs at different HCl volumes from 0.021 to 0.060 mL. The spectra show the formation of separate plasmonic resonance peaks as the volume increases due to the formation of different anisotropic shapes [42]. The increase in HCl volumes from 0.036 to 0.056 mL showed a second-order polynomial relationship between the increasing volume and the LSPR peaks with a bathochromic shift from 592 to 750.2 nm and a regression coefficient of 0.9858 (Figure 2B). This relationship has also been seen in binary surfactant AuNRs synthesis [14]. However, the bathochromic shift of the transversal surface plasmonic resonance (TSPR) peak was also observed when HCl volumes increased from 0.050 mL to 0.060 mL with a shift from 544 to 579. In addition, the TSPR peak at 0.060 mL HCl volume showed a broad peak, which overlapped with the LSPR peak at 715 nm due to the high formation of bigger spherical nanoparticles at this volume. The HCl concentration below 0.036 mL was too low to accelerate the growth kinetics, while 0.056 mL is the maximum HCl volume needed to accelerate the growth kinetics, leading to rod formation. The mechanism behind this could be attributed to the fact that at a smaller amount of HCl, HQ becomes oxidized to the semiquinone radical, which slowly reduces Au(I) to Au (0). When the HCl volume is at the maximum (0.054 mL), the HQ is further oxidized to quinone. This increased reaction rate results in a high yield of bigger spheres. The effect of the seed solution was investigated by increasing its volume from 0.04 to 2 mL and keeping all parameters constant. Figure 2C shows that increasing the seed solution volume in the growth solution favors a bathochromic shift of the LSPR peak from 592 to 792.5 nm. The increases in seed solution favored the increase in the aspect ratio (Figure 2C, greyline), even though the length and diameter of the rods were not the same. The decrease in the length was observed with the increasing seed solution (Figure 2G).

**Figure 3:**
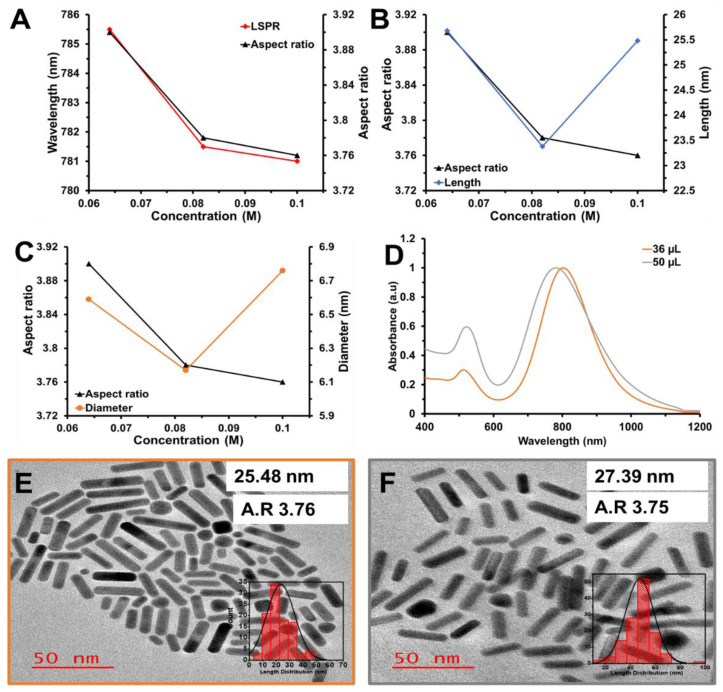
The relationship between HQ concentration and (**A**) LSPR band wavelengths and aspect ratio, (**B**) aspect ratio and rod length, and (**C**) aspect ratio and rod diameter. (**D**) Normalized UV-Vis-Vis spectra of AuNRs synthesized with 0.1 M HQ and, 0.050 mL, and 0.036 mL HCl with constant AgNO_3_ concentration and seed solution. TEM images of AuNRs with 0.1 M HQ and (**E**) 0.050 mL or (**F**) 0.036 mL HCl, under constant AgNO_3_ concentration and seed solution.

Increasing the HQ concentration from 0.064 to 0.1 M with 0.50 mL of HCl (12.1 M) and 2.0 mL of the seed solution showed a slight blue-shift of the LSPR from 785.5 to 781 nm with a decrease in the aspect ratio (Figure 3A). The rods’ length or width is proportional to the increased HQ concentration (Figure 3B,C). We further evaluated the effect of decreasing the HCl volume from 0.05 mL to 0.036 mL, using 0.1 M HQ and 2 mL of seed solution. A red-shifted LSPR peak was observed from 781 to 804 nm (Figure 3D). This bathochromic shift could be attributed to the favorable pH conditions that allowed mild activation of the HQ reduction process of Au(I) to Au (0), leading to the increasing length. This can be observed from the TEM results, which show an expanding size of rods from 25.48 ± 8.4 nm × 6.76 ± 1.2 nm to 27.39 ± 7.9 nm × 7.31 ± 1.6 nm with a slight increase in the width (Figure 3E,F). At optimum conditions of 0.036 mL (HCI volume), 0.1 M HQ, and a 2 mL seed solution, a solution with 87.7% rod-shaped particles was achieved. In comparison with the existing literature, this was the first mini-AuNRs absorbing at 800 nm synthesized from the binary-surfactant seed-mediated method with the smallest size (Table 1).

**Figure 4:**
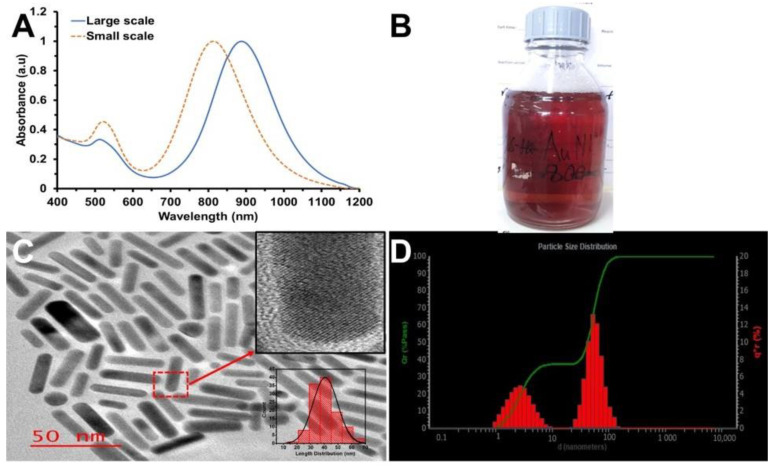
(**A**) UV-Vis-NIR spectra of large- and small-scale synthesized AuNRs. (**B**) Photographic image of large-scale (500 mL) synthesized AuNRs. (**C**) TEM image of AuNRs synthesized at a large scale (Scale: 50 nm); inset: HRTEM image of large-scale synthesized AuNRs. (**D**) DLS size distribution graph of large-scale synthesized AuNRs.

The AuNRs’ synthesis with 0.036 mL HCl was scaled up to 500 mL. The absorption spectra showed a bathochromic shifting of the LSPR peak by 83 nm at a large-scale synthesis compared to the small-scale synthesis, as shown in Figure 4A. Figure 4B shows the photographic image of the 500 mL synthesized AuNRs. The shape and size analysis showed rod-shaped particles with a size range of 40.70 ± 9.1 nm (Figure 4C). The synthesis was repeated many times, and the average sizes and LSPR peak were the same. The inset in Figure 4C is the HR-TEM image of the AuNRs, showing the crystal pattern of the AuNRs. The hydrodynamic dimension from the DLS for the large-scale synthesized AuNRs showed similar results to TEM. However, the diameter was smaller than the TEM size distribution. The DLS results obtained were 49.9 × 2.4 nm (Figure 4D). According to the literature, the small-sized peak in the DLS is not truly the diameter of the rods but a diffusion rotation coefficient of the non-spherical particles in the rod solution, and it is equivalent to the translation diffusion coefficient of a spherical particle with an average diameter of 2.4 nm [44].

**Figure 5:**
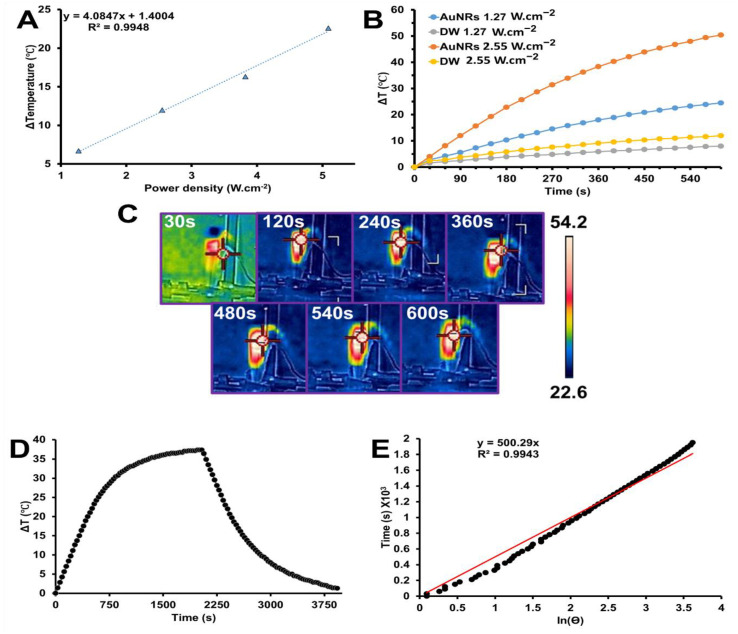
(**A**) Evaluation of different laser power densities (1.27, 2.55, 3.82, and 5.09 W·cm^−2^) using 808 nm in water. (**B**) Temperature change elevation of the pure water and the aqueous dispersion of AuNRs with different laser power densities (1.27 and 2.55 W·cm^−2^) as a function of irradiation time (0–10 min) at ambient temperature. (**C**) IR camera images of AuNRs irradiated with 808 nm Laser at 2.55 W·cm^−2^ for 10 min (**D**) The photothermal effect of the aqueous dispersion of the AuNRs (OD:0.160) irradiated with 808 lasers (a power density of 1.27 W·cm^−2^), and then the laser was shut off. (**E**) The time constant for heat transfer from the system is determined to be τs = 505.7 s by applying the linear time data from the cooling period (after 1830 s) versus the negative natural logarithm of driving force temperature.

AuNRs have been used for photothermal applications such as cancer therapy, as well as antimicrobial and therapeutic applications; therefore, it is worth testing the photothermal conversion of the as-synthesized AuNRs. Different laser power densities (1.27, 2.55, 3.82, and 5.09 W·cm^−2^) were evaluated using 808 nm in water. The temperature change increased from 6.7 to 12.0, 16.3, and 22.6 °C with the increasing power density after 630 s irradiation with a linear regression (R^2^) of 0.99 (Figure 5A). From the results, the photothermal profile of AuNRs was evaluated using the less heat-producing power density 1.27 and 2.55 W·cm^−2^. The AuNRs produced heat up to 24.5 and 50.4 °C after irradiating them for 630 s (Figure 5B). The IR camera images in Figure 5C confirm the heat production of AuNRs at different times during the irradiation (2.55 W·cm^−2^) with a maximum temperature of 54.2 °C. The photothermal conversion efficiency was calculated after illuminating the AuNRs with 1.27 W·cm^−2^ by irradiating them until reaching saturation temperature and allowing them to cool down to the initial temperature (Figure 5D). The τ_s_ was found to be 500.29 s by applying the linear time data from the cooling period (after 1830 s) versus the negative natural logarithm of the driving force temperature (Figure 5E). The photothermal conversion of the as-synthesized AuNRs at a power density of 1.27 W/cm^2^ is 20.86%, which is similar to other reported AuNRs’ photothermal conversion efficiency [45,46,47].

## 4. Conclusions

The AuNRs were successfully synthesized using a binary surfactant seed-mediated method with HQ as the reducing agent. The effect of the Ag concentration, HQ concentration, HCl volumes, and seed solution volumes on the morphology and optical property of the as-synthesized AuNRs was investigated using TEM and UV-Vis-NIR spectroscopy. The UV-Vis-NIR spectra showed an increasing LSPR peak position from 592 to 750.2 nm as the HCl volume increased from 0.036 to 0.056 mL. By adjusting the seed solution volume in the growth solution, the LSPR can be further tuned to 792.5 nm. Decreasing the HQ concentration resulted in a blue shift in the LSPR wavelength with an increasing aspect ratio from 3.76 ± 0.7 to 3.78 ± 0.8. AuNRs absorbing at 800 nm were obtained by adjusting the HCl volume to obtain mini-AuNRs with an average size of 27.39 ± 7.9 nm × 7.31 ± 1.6 nm and shape percentage yield of 87.7% while maintaining the aspect ratio. In addition, the synthesis was successfully upscaled to 500 mL. However, the UV-Vis-NIR spectra of the large-scale AuNRs showed a bathochromic shifting of the LSPR peak by 83 nm compared to the small-scale AuNRs with an average length size of 40.70 ± 9.1 nm. The as-synthesized AuNRs produced a photothermal conversion efficiency of 20.86% upon irradiation with an 808 nm laser at a power density of 1.27 W·cm^−2^, which showed that the as-synthesized AuNRs could be used as a photothermal agent.

## Figures and Tables

**Table 1:** The comparison table of AuNRs’ size parameters (length, diameter, and aspect ratio) absorbing at ~800 nm from different seed-mediated syntheses.

Surfactant	Reducing Agents	Size (nm)	AR	Ref.
CTAB (0.037 M) and NaOL (0.0126 M)	AA	97.2 ± 4.9 × 25.1 ± 1.2	3.87	[14]
CTAB (0.01 M) and NaOL (0.005 M)	AA	71.7 ± 9.2 × 20.9 ± 2.3 and 87.7 ± 10.1 × 23.7 ± 2.2	3.5 ± 0.7 and 3.7 ± 0.5	[40]
CTAB (0.1 M)	AA and HQ	21.7 ± 5.5 × 5.8 ± 0.8 and 27.2 ± 4.4 × 5.0 ± 0.5	3.8 ± 1.0 and 5.6 ± 1.3	[20]
CTAB (0.1 M)	AA	36.6 ± 4.0 × 8.8 ± 0.6	4.3 ± 0.7	[43]
CTAB (0.037 M) and NaOL (0.0126 M)	HQ	27.39 ± 7.9 nm × 7.31 ± 1.6 nm	3.75 nm	This work

## Data Availability

The data presented in this study are available on request from the corresponding author.
